# Genetic characterization of juvenile sudden cardiac arrest and death in Tuscany: The ToRSADE registry

**DOI:** 10.3389/fcvm.2022.1080608

**Published:** 2022-12-14

**Authors:** Francesca Girolami, Valentina Spinelli, Niccolò Maurizi, Martina Focardi, Gabriella Nesi, Vincenza Maio, Rossella Grifoni, Giuseppe Albora, Bruno Bertaccini, Mattia Targetti, Raffaele Coppini, Silvia Favilli, Iacopo Olivotto, Elisabetta Cerbai

**Affiliations:** ^1^Cardiology Unit, Meyer Children’s University Hospital, Florence, Italy; ^2^Department of Neurosciences, Psychology, Drug Research and Child Health, University of Florence, Florence, Italy; ^3^Careggi University Hospital, Florence, Italy; ^4^Forensic Medical Sciences, Department of Health Sciences, University of Florence, Florence, Italy; ^5^Division of Pathological Anatomy, Department of Health Sciences, University of Florence, Florence, Italy; ^6^Department of Statistics, Computer Science, Applications, University of Florence, Florence, Italy; ^7^Department of Experimental and Clinical Medicine, University of Florence, Florence, Italy

**Keywords:** juvenile sudden cardiac death, ToRSADE registry, molecular autopsy, next generation sequencing, genetics, arrhythmia

## Abstract

**Background:**

Sudden cardiac arrest (SCA) in young people represents a dramatic event, often leading to severe neurologic outcomes or sudden cardiac death (SCD), and is frequently caused by genetic heart diseases. In this study, we report the results of the Tuscany registry of sudden cardiac death (ToRSADE) registry, aimed at monitoring the incidence and investigating the genetic basis of SCA and SCD occurring in subjects < 50 years of age in Tuscany, Italy.

**Methods and results:**

Creation of the ToRSADE registry allowed implementation of a repository for clinical, molecular and genetic data. For 22 patients, in whom a genetic substrate was documented or suspected, blood samples could be analyzed; 14 were collected at autopsy and 8 from resuscitated patients after SCA. Next generation sequencing (NGS) analysis revealed likely pathogenetic (LP) variants associated with cardiomyopathy (CM) or channelopathy in four patients (19%), while 17 (81%) carried variants of uncertain significance in relevant genes (VUS). In only one patient NGS confirmed the diagnosis obtained during autopsy: the p.(Asn480Lysfs*20) PKP2 mutation in a patient with arrhythmogenic cardiomyopathy (AC).

**Conclusion:**

Systematic genetic screening allowed identification of LP variants in 19% of consecutive patients with SCA/SCD, including subjects carrying variants associated with hypertrophic cardiomyopathy (HCM) or AC who had SCA/SCD in the absence of structural cardiomyopathy phenotype. Genetic analysis combined with clinical information in survived patients and post-mortem evaluation represent an essential multi-disciplinary approach to manage juvenile SCD and SCA, key to providing appropriate medical and genetic assistance to families, and advancing knowledge on the basis of arrhythmogenic mechanisms in inherited cardiomyopathies and channelopathies.

## Introduction

Among all cases of out-of-hospital sudden cardiac arrest (SCA) or sudden cardiac death (SCD), those occurring at juvenile age are matters of social and clinical urgency. SCD is defined as a natural and unexpected cardiovascular collapse within 1 h of initial acute symptoms (1), affecting apparently healthy young people (≤ 40 years). SCD requires carefully designed, regional, or multicenter studies to generate accurate statistics regarding incidence and risk factors (2). In Italy, the incidence of SCD in the general population is 1/1,000 person/year (3) although in younger individuals the value is much lower (around 1/100,000 per year). These numbers are necessary to formulate useful public health policy for the early detection and prevention. In subjects below 35 years of age, no cause is identified in up to 30% of cases after clinical autopsy (4). Because major etiologies of SCD in the young are associated with inherited conditions, a genetic characterization and management of family members must often be conducted. Case series of SCD are valuable for defining etiology and mechanisms but they cannot quantify the denominator population (2). The creation of well-designed clinical registries is an emerging worldwide necessity; recently, many registries have been developed to monitor the occurrence of SCD/SCA in the young. SCD/SCA registries already created (49 of SCA and 15 of SCD) have been shown to support epidemiological analysis and ameliorate the attention between ambulance services, hospital procedures and clinical/molecular autopsy (4–10). In a pilot anonymized research study promoted by the Tuscany region, we outlined an algorithm suitable to monitor, collect, and investigate SCA or SCD in people aged from 18 to 50 years, referred to hospitals of the Florentine area.

The project planned to create and exploit the ToRSADE (Tuscany Registry of Sudden Cardiac Death) registry to (i) identify and record all patients deceased from SCD or resuscitated after SCA in Tuscany, (ii) establish a diagnostic-therapeutic plan and preventive-research strategy against SCD or SCA events in family members. We here describe the clinical records collected at Careggi University Hospital during the period 2016–2019.

## Methods

### Study cohort and clinical data

The study was approved by the Local Ethical Committee (No. BIO.16.011) and conformed to the ethical guidelines of the declaration of Helsinki. The registry was based on the following inclusion and exclusion criteria.

#### Inclusion criteria

– Age < 50 years;
– Patients who died in hospital due to brain death following cardiac arrest in whom autopsy was performed;
– Patient who died due to out-of-hospital cardiac arrest and in whom forensic autopsy was performed;
– Patients who survived cardiac arrest.

#### Exclusion criteria

– Patients surviving cardiac arrest who denied informed consent;
– In-hospital or out-of-hospital death when cardiac arrest occurred as a consequence of traumatic injuries, toxicological causes, neoplastic or infectious disease and structural cardiac abnormalities.

Resuscitated patients were transferred to the emergency department of Careggi University Hospital for post-cardiac arrest care and clinical data collected where possible; blood samples were used for genetic testing. SCD subjects underwent full autopsy as described below.

### Autopsy and histology studies

According to the 2017 guidelines for autopsy investigation of SCD developed by the association for European cardiovascular pathology (AECVP) (11), a complete external and internal post-mortem examination was performed in all cases. The hearth was taken and fixed in formalin for subsequent examination. Evaluation of the conduction system, using a simplified method, was performed when no pathological findings were observed at gross or histological examination (11, 12). Myocardial samples of the ventricles and the major epicardial coronary arteries were then included in paraffin and cut with a microtome in micrometric slides. Hematoxylin and eosin staining was performed and observed under a microscope. If required, we also performed additional histological stains. Toxicological investigations were undertaken on peripheral venous blood. Finally, we sampled and stored blood (at −20°) and fresh tissues (heart, liver, and spleen at −80°) for long-term preservation.

### Genetic analysis using Next Generation Sequencing (NGS)

A panel of 174 cardiac genes implicated in cardiomyopathies or channelopathies was analyzed by Next Generation Sequencing (NGS). Specifically, genomic DNA was automatically extracted from whole-blood samples, using QIAsymphony DSP DNA Kits in combination with the QIAsymphony SP (Qiagen, Hilden, Germany), following the manufacturer’s protocol. Gene libraries were prepared from DNA using an Illumina Nextera TruSight™ Cardio Sequencing Kit (Illumina Inc., San Diego, CA, USA). The sequencing step was performed on an Illumina MiSeq system, targeting for 151 bp pair-end reads, and a mean sequencing coverage averaging above 200×. Variant analysis was performed by Local Run Manager and Base Space Variant Interpreter software (Illumina Inc., San Diego, CA, USA). Sanger sequencing was used to confirm clinically relevant detected variants (forward and/or reverse strand). All variants were classified according to the American college of medical genetics (ACMG) classification (13) and the association for clinical genomic science (ACGS) (14, 15). Reanimated subjects received genetic counseling and gave informed consent before performing genetic tests (see Supplementary material).

**FIGURE 1:**
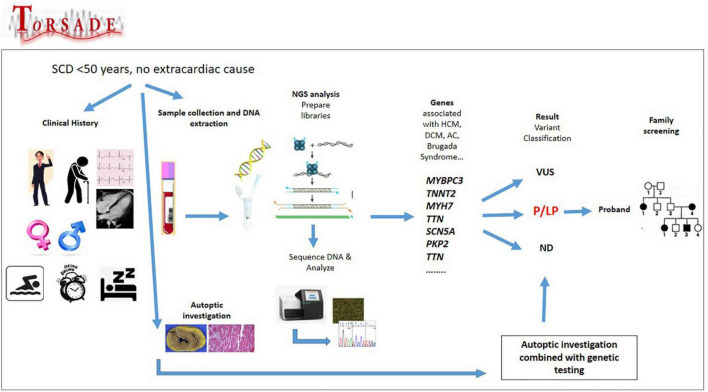
Genetic characterization of ToRSADE patients: an overview of ToRSADE workflow for the genetic characterization of diseases involved in sudden cardiac death.

The Figure 1 shows the genetic characterization of ToRSADE patients.

## Results

Between July 2016 and February 2019, of 52 SCA/SCD victims seen at Careggi University Hospital (AOUC), 22 subjects met the inclusion criteria (15–18). Eight resuscitated patients underwent genetic analysis; the remaining 14, who died out of hospital or were brain-dead after SCA underwent clinical autopsy and genetic testing. For comparison, 34 subjects < 50 years were filed as out-of-hospital SCD by the local rescue service over the same period of time and referred to the district hospitals; however, none of them was genotyped and only three were annotated as receiving autopsy, but no results were available. The main features of the 22 study subjects are shown in Table 1. Data regarding past medical history and family history were available only for survived patients (Table 2). In 1 out of 14 cases, autopsy led to a diagnostic hypothesis of arrhythmogenic cardiomyopathy (AC) but, in the majority of cases, autoptic results were uncertain or inconclusive. Genetic investigation identified four likely pathogenic (LP) variants in deceased patients (submitted in ClinVar database^1^). Overall, 3/4 LP variants (75%) were found in genes associated with hypertrophic cardiomyopathy (HCM) (MYBPC3 c. 2441_2443del ClinVar ID#177700, TNNT2 c.517_519del ClinVar ID#43648, and TCAP c.360_361del ClinVar ID#1708038), one was detected in PKP2 c. 1440_1444del (ClinVar ID# 419977) associated with AC. Variants of uncertain significance (VUS) were found in 17 cases (eight resuscitated and nine dead) in genes associated with HCM or dilated cardiomyopathy (DCM), such as TTN, TNNT2, MYH6, DSP, ACTN2, CALR3, and MYH7. Of note, one SCD victim carried the SCN5A variant c.1398G > T (ClinVar ID#201453) associated with Brugada syndrome and classified as a VUS based on the ACGS guidelines (13, 14).

**TABLE 1:** Genetic findings in the Tuscany registry of sudden cardiac death (ToRSADE) registry.

Case	Age	Circumstance of death	Molecular autopsy or subsequent cardiological treatment	Autoptic or clinical diagnosis	Pathogenic evidence according to ACMG and ACGS standards. Predicted effect of the variant.	Affected gene	Nucleotide change	Amino acid change	Disease classification related to affected gene	Evidence for previously reported variants
#2	20	Football match	Clinical autopsy	N/C	LP (PM1_strong, PP3_moderate, PM2_supporting, PP5_supporting) Inframe-deletion	MYBPC3	c.2441_2443del	p.(Lys814del)	Hypertrophic cardiomyopathy	PMID: 12110947, 16566404, 18957093 ClinVar variation ID: 177700 HGMD: CD021840
#15	47	At rest	Clinical autopsy	N/C	LP (PM2_moderate, PM4_moderate, PS4_strong) Inframe-indel	TNNT2	c.517_519del	p.(Glu173del)	Hypertrophic cardiomyopathy	PMID: 7898523, 27036851, 10731693 ClinVar variation ID: 43648 HGMD: CD951865
#18	35	At rest	Clinical autopsy	Arrhythmogenic cardiomyopathy	LP (PVS1_very strong, PM2_moderate) Frameshift	PKP2	c.1440_1444del	p.(Asn480Lysfs*20)	Arrhythmogenic cardiomyopathy	ClinVar variation ID: 419977 HGMD: CD148578
#19	28	At rest	Clinical autopsy	N/C	LP (PS1_strong, PM2_moderate) Frameshift	TCAP	c.360_361del	p.(Glu120Aspfs*15)	Cardiomyopathy, hypertrophic, dilated cardiomyopathy, Muscular dystrophy, limb-girdle, autosomal recessive	HGMD: CD189693
#4	38	N/A	Resuscitated after cardiac arrest	N/A	VUS	MYBPC3	c.2381C > T	p.(Pro794Leu)	Hypertrophic cardiomyopathy	ClinVar variation ID: 180968 HGMD: CM1714728
#12	47	At rest	Clinical autopsy	N/C	VUS	TTN (NM_001267550)	c.10852C > T	p.(Gln3618*)	Dilated cardiomyopathy	ClinVar variation ID: 223254
#14	N/A	N/A	Resuscitated after cardiac arrest	N/A	VUS	MYH7	c.161G > A	p.(Arg54Gln)	Hypertrophic cardiomyopathy	ClinVar variation ID: 42856
#17	38	At rest	Clinical autopsy	N/C	VUS	MYH7	c.2890G > C	p.(Val964Leu)	Hypertrophic cardiomyopathy, dilated cardiomyopathy	PMID: 19412328, 23349452, 24704860 ClinVar variation ID: 42938 HGMD: CM087588
#1	24	At rest	Clinical autopsy	N/C	VUS	MYH6	c.3010G > T	p.(Ala1004Ser)	SIDS, dilated cardiomyopathy, hypertrophic cardiomyopathy, atrial septal defect	PMID: 24119082, 22361390 HGMD: CM052257
						RAF1	c.122G > A	p.(Arg41Gln)	Rasopathy/Noonan spectrum disorder	ClinVar variation ID: 40586
#3	56	At rest	Resuscitated after cardiac arrest	N/C	VUS	DSP	c.3979A > G	p.(Ile1327Val)	Carvajal syndrome, dilated cardiomyopathy	None
#5	33	At rest	Clinical autopsy	N/C	VUS	AKAP9	c.2276T > C	p.(Met759Arg)	Ventricular fibrillation/ LQT, Romano Ward syndrome	None
						MYH6	c.4974G > T	p.(Gln1658His)	Dilated cardiomyopathy, hypertrophic cardiomyopathy, atrial septal defect	
#6	N/A	N/A	Resuscitated after cardiac arrest	N/A	VUS	TRPM4	c.3620del	p.(Pro1207Leufs*26)	Progressive familial heart block, Brugada syndrome, SIDS	None
						PKP2	c.2020G > A	p.(Val674Met)	Arrhythmogenic cardiomyopathy	ClinVar variation ID: 464418
						MYH7	c.319G > T	p.(Asp107Tyr)	Hypertrophic cardiomyopathy	None
						KCNH2	c.446G > T	p.(Gly149Val)	Long_QT syndrome	None
#7	34	At work	Resuscitated after cardiac arrest	N/A	VUS	MYLK	c.76T > C	p.(Ser26Pro)	Aortic aneurysm, familial thoracic	None
#8	35	During sport activities	Clinical autopsy	N/C	VUS	SCN5A	c.1398G > T	p.(Leu466Phe)	Brugada syndrome, LQT, dilated cardiomyopathy	ClinVar variation ID: 201453 HGMD: CM146794
#9	49	N/A	Resuscitated after cardiac arrest	N/A	VUS	CALR3	c.166T > C	p.(Phe56Leu)	Hypertrophic cardiomyopathy	None
#10	N/A	At rest	Resuscitated after cardiac arrest	N/A	VUS	MYO6	c.427A > G	p.(Met132Val)	Deafness, with hypertrophic cardiomyopathy	None
#11	28	At rest	Clinical autopsy		VUS	ACTN2	c. 1823G > A	p.(Arg608Gln)	Hypertrophic cardiomyopathy	None
						DSP	c.2723G > A	p.(Arg908His)	Carvajal syndrome, dilated cardiomyopathy	ClinVar variation ID: 177781 HGMD: CM114459
#13	50	At rest	Resuscitated after a cardiac arrest event	N/A	VUS	ANK2	c.533C > T	p.(Ala178Val)	Long QT syndrome	None
						SDHA	c. 1090G > A	p.(Val354Ile)	Hypertrophic cardiomyopathy	ClinVar variation ID: 412322
						MYH6	c.2540C > T	p.(Thr847Met)	Dilated cardiomyopathy, hypertrophic cardiomyopathy	ClinVar variation ID: 312869
#16	47	At rest	Clinical autopsy	N/C	VUS	CBS	c.1105C > T	p.(Arg369Cys)	Familial thoracic aortic aneurysm	None
#20	49	At rest	Clinical autopsy	N/C	No variants detected	–	–	–	–	None
#21	N/A	N/A	Clinical autopsy	N/C	VUS	TNNT2	c.113C > T	p.(Ala38Val)	Dilated cardiomyopathy, hypertrophic cardiomyopathy, left ventricular non-compation	ClinVar variation ID: 43675
#22	N/A	N/A	Clinical autopsy	N/C	VUS	TTN	c.57515_57517del	p.(Thr19172del)	Dilated cardiomyopathy, hypertrophic cardiomyopathy	None

LP, likely pathogenic; LQT, long QT interval; VUS, variant of uncertain significance; SIDS, sudden infant death syndrome; N/C, not conclusive; N/A, not available. ClinVar = https://www.ncbi.nlm.nih.gov/clinvar/ HGMD Professional = http://www.hgmd.cf.ac.uk/docs/login.html.

**TABLE 2:** Data regarding past medical history and family history of Tuscany registry of sudden cardiac death (ToRSADE) survived patients.

Case	Medical history	Family history	Diagnostic tests performed after cardiac arrest and treatment
#3	Signs and symptoms: supraventricular ectopic beats Pharmacological treatment: none Other diseases: none	Negative	ECG: supraventricular ectopic beats. QTc 470 m s ICD implantation
#4	Signs and symptoms: syncope Pharmacological treatment: cardicor, amiodarone, edoxaban Other diseases: keratoconus	Both parents affected by atrilal fibrillation Proband’s father affected by dilated cardiomyopathy	ECG: AVB I degree, TWI D3 Echo: posterior mitral leaflet prolapse ICD implantation
#6	Signs and symptoms: obesity, dyslipidemia Pharmacological treatment: amiodarone, bisoprolol, ramipril, lixiana Other diseases: none	Negative	ECG: SR, QS in inferior leads; TWI V3–V6 and in inferior leads RMN: EF 38%, thinning of lateral LV wall with some non-compaction and DE subepicardial ICD implantation
#7	Signs and symptoms: cardiac surgery for thoracic aortic aneurysm valve sparing (David operation) Pharmacological treatment: bisoprolol Other diseases: psychiatric disorder	Proband’s mother with suspected Marfan syndrome	Angio TC: negative for dissection ECG: LongQT S-ICD implantation
#9	Signs and symptoms: none Pharmacological treatment: bisoprolol Other diseases: none	Negative	ECG: rSR’s in right precordial leads RMN: negative ICD implantation
#10	Signs and symptoms: mitral prolapse, an episode of tachycardia Pharmacological treatment: none Other diseases: none	Both parents affected by hypertension. Mitral valve prolapse known since age 19, cardiac surgery refused.	ICD not implanted due to neurological state (postanoxic encephalopathy)
#13	Signs and symptoms: none Pharmacological treatment: none Other diseases: none	Negative	None
#14	Signs and symptoms: Cardiac arrest at rest Pharmacological treatment: none Other diseases: none	Negative	ECG: rSR’s in V1, TWI in inferior leads and V3–V4. Coronarography negative. RMN not performed. ICD not implanted due to severe postanoxic encephalopathy.

ECG, electrocardiogram; Echo, echocardiogram; ICD, implantable cardioverter-defibrillator; RMN, cardiac magnetic resonance; angio TC, computed tomographic angiography.

### Combined autoptic and genetic investigations

**FIGURE 2:**
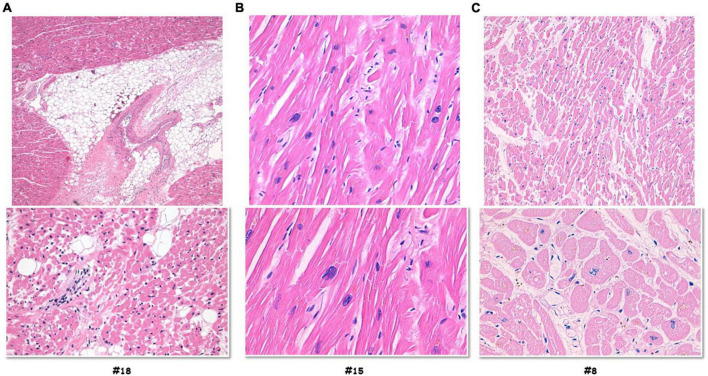
Histology investigation of three selected sudden cardiac death cases. Hematoxylin and eosin staining performed on myocardial samples of the ventricles. Case #18, **(A)** histology showed myocytes loss and disorganization with replacement of fibro-fatty tissue; myocardial inflammatory infiltrates were present, but granulomas or giant cells were absent. Case #15, **(B)** the images showed myocytes disarray with storiform pattern, and occasionally myocytes exhibiting nuclear enlargement and hyperchromasia. Masson trichrome stain displayed interstitial and replacement-type fibrosis. Case #8, **(C)** analysis of conduction system and ventricular tissue did not reveal microscopic anomalies or inflammatory changes.

**FIGURE 3:**
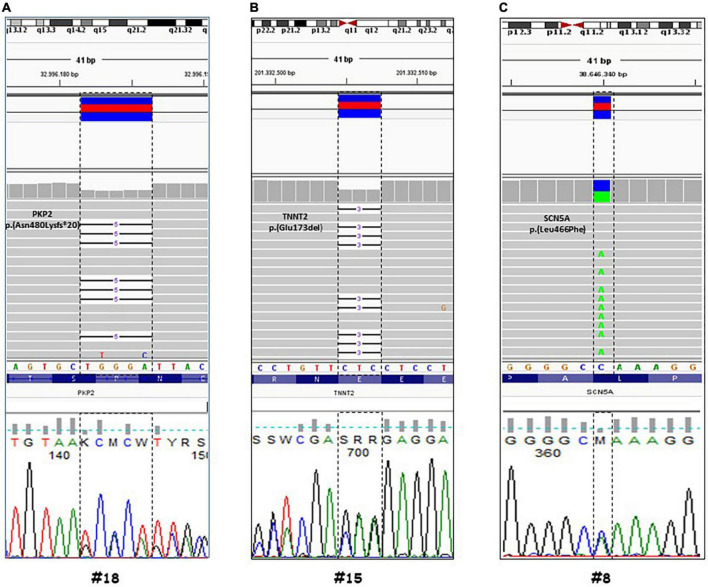
Genetic analysis by next generation sequencing (NGS) of three selected cases. Visualization by integrative genomic viewer (IGV) software of NGS data (through alignment of enriched sequences to Hg19). Case #18, **(A)** a heterozygous deletion in the PKP2 gene (Genbank accession no. NM_004572.3), c.1440_1444del p.(Asn480Lysfs*20). Case #15, **(B)** a deletion in TNNT2 (Genbank accession no. NM_000364.2), c.517_519del p.(Glu173del) in heterozygous state. Case #8, **(C)** a missense variant in the SCN5A gene (Genbank accession no. NM_198056), c.1398G > T p.(Leu466Phe). The detected variants were confirmed by Sanger sequencing.

In 12 out of 14 cases, the autopsy results were inconclusive; however, combining autopsy and genetic testing allowed a definitive diagnosis in a 35-year-old woman (patient #18) who collapsed and died while at rest. At autopsy, cardiovascular examination showed a 350-g heart with multifocal areas of wall thinning and apparent fatty infiltration, ventricular thickness being within normal values. Histologically, myocytes loss and disorganization with replacement of fibro-fatty tissue was observed (Figure 2A). Myocardial inflammatory infiltrates were present, but granulomas or giant cells were absent. These findings were consistent with AC. Genetic testing detected a heterozygous deletion in the PKP2 gene (Genbank accession no. NM_004572.3), c.1440_1444del p.(Asn480Lysfs*20) (Figure 3A). This truncated variant has been previously described in patients with AC (19), annotated in ClinVar and in human gene mutation professional databases. The genome aggregation database (gnomAD)^2^ has reported this variant with an allele frequency of 0.00001105 in the general population. According to ACMG and ACGS guidelines (13, 14), this variant is classified as LP. Patient #15 was a 47-year-old man found unconscious outside his house. On arrival, the medical team proceeded to cardiopulmonary resuscitation but without success. At autopsy, external examination showed a lean male of 186 cm in height and 71 kg in weight. There was no evidence of external injuries. Internal examination revealed a 480-g heart with a body mass index (BMI) of 20.5. The thickness of the right and left ventricular walls was 5 and 15 mm, respectively. There was no sign of acute myocardial infarction. Histology showed myocyte disarray and myocytes exhibiting nuclear enlargement and hyperchromasia were occasionally seen. Masson trichrome stain displayed interstitial and replacement-type fibrosis (Figure 2B). Genetic analysis revealed a deletion in TNNT2 (Genbank accession no. NM_000364.2), c.517_519del p.(Glu173del) in heterozygous state (Figure 3B). This variant has been previously described in patients with HCM (20–22), annotated in ClinVar database and human gene mutation professional database, but not reported in the gnomAD (see text footnote 2). Since multiple tools of computational evidence support a deleterious effect on the gene or protein, the variant was classified as LP according to current ACMG and ACGS guidelines (13, 14). Finally, we report a case where a possible diagnosis was made solely on the basis of the genetic test. Patient #8 was a 35-year-old male professional athlete who died suddenly while swimming. At autopsy, both lungs appeared swollen and multi-organ congestion was evident. The heart (weight: 450 g; LV thickness: 1.2 cm; IVS thickness: 2.0 cm; RV thickness: 0.5 cm) showed no relevant gross findings. Similarly, analysis of conduction system and ventricular tissue did not reveal microscopic anomalies or inflammatory changes (Figure 2C). Genetic analysis showed a missense variant in the SCN5A gene (Genbank accession no. NM_198056), c.1398G > T p.(Leu466Phe) (Figure 3C). This variant is annotated in ClinVar and in human gene mutation professional databases in association with Brugada syndrome (23). The gnomAD (see text footnote 2) has reported this variant with an allele frequency of 0.00002413 on a total population and multiple tools of computational evidence supporting a deleterious effect on the gene or protein. The variant was classified as a VUS (13, 14).

## Discussion

Our study represents a systematic approach aimed at exploring the feasibility and relevance of a regional registry of SCD in Tuscany, starting from a defined geographical area (Florence) and during a 30-month pilot regional project. In our experience, the estimated incidence of juvenile sudden death was approximately 1/100,000 per year, in line with the incidence reported by Del Vecchio and Padeletti (3); there was a prevalence of male subjects (14 vs. 8) (24), and mean age was 39 years. Among the 22 victims, autopsy (in 14 victims) and clinical data in survived patients failed to identify the underlying disease in most. Likewise, subsequent genetic tests was often inconclusive. A negative genetic test is not surprising given the lack of definite genetic diagnosis in over 50% of patients with clinically symptomatic, likely inherited cardiomyopathies (25). In a recent study from our institution based on over 1,200 HCM patients, the contemporary yield of genetic screening is 47% (26). Notably, however, in the current study NGS identified four LP variants, mostly involved genes causing structural cardiomyopathies: all had a negative autopsy except one patient with evidence of AC. Overall, 19% of patients exhibited a clinically actionable variant, comparing favorably with the diagnostic yield of molecular autopsy in the literature, ranging from 20 to 35% (27). VUS were found in 17 cases. These inconclusive genetic testing results may be due to the bias to perform variants interpretation without a clear diagnosis and a clear pertinence of the phenotype to the affected gene and with a negative or inconclusive autopsy. In the process of genetic test interpretation the clinical diagnosis has a key role because represents one of the most important criteria (PP4 according to ACMG guidelines for variants classification) (13). Indeed, in the case of victims of SD the genetic result may be often inconclusive. In our study we did not report VUS likely benign or benign instead we have annotated VUS potentially pathogenic. To investigate the pathogenic role of the VUS identified we could perform *in vivo* or *in vitro* functional studies supportive of a damaging effect on the gene. In addition these VUS variants should be periodically revaluated on the basis of new literature/public databases data (i.e., ClinVar, PubMed, HGMD, Clin Gen) (13). In one case, with negative autopsy, genetic testing revealed the p.(Leu466Phe) variant in SCN5A, associated with Brugada syndrome (28). Notably, in four subjects carrying LP variants in genes associated with HCM (MYBPC3, MYH7 and TCAP), SCD occurred in the absence of clear structural disease manifestation at autopsy. Recent studies highlighted that cardiomyocyte functional abnormality, occurring as a direct consequence of sarcomere mutations, may occur before the onset of gross hypertrophy or dilatation, and may theoretically lead to increased arrhythmic risk in mutation carriers in the absence of hypertrophy (29, 30). Nevertheless, a clear link between these mutations and the SCA/SCD event is elusive, while arrhythmic risk seems particularly low in sarcomere mutation carriers without an HCM phenotype. Therefore, the clinical value of this anecdotal observation requires further investigation in larger series (31). All in all, our study supports the idea that genetic test represents a significant source of information to infer the cause of death, or resuscitated cardiac arrest, in otherwise healthy young people. Building of these promising observations, the use of more extended genetic analyses in genotype-negative SCA/SCD victims (exome, whole genome), paralleled by targeted *in vitro* studies, may offer further insight into the substrates of juvenile arrhythmias. From a public health perspective, the creation of a local but expandable registry, such as ToRSADE for Tuscany, allows clinicians to plan the clinical and genetic screening strategy for potentially affected relatives, both in the presence of a clear autoptic diagnosis or of non-conclusive autoptic results (3). Since the ToRSADE project was based on an anonymous registry, cascade family screening was not feasible at this stage. However, these results have led to implement a prospective clinical enrolment protocol, now active at our hospital, which now allows such policy. Partnering with a similar initiative in Pisa (The JUST project “JUvenile Sudden cardiac deaTh: JUST know and treat”), the plan is to extend this algorithm to the whole Tuscany region. At variance with previous studies performed in larger groups (32), we performed genetic testing only in subjects where clinical examination or autopsy excluded coronary artery disease. This is of particular relevance for two reasons. First, the number of LP variants retrieved in our samples was due to the pre-selection of cases of unexplained cardiac death excluding toxicological, infectious, oncological, trauma conditions or structural cardiac abnormalities. Second, the presence of cases of interest older than 40 sheds light on the occurrence of arrhythmic cardiac arrest due to non-ischemic causes in subjects usually excluded from epidemiology of juvenile SCD. ToRSADE is the first Tuscan registry to combine forensic and non-forensic autopsy, histology, clinical data evaluation (available only for survived patients) and genetic testing of SCA patients and SCD victims. Despite the time and geographic limitations, the rigorous multidisciplinary approach allowed identifying variants deserving further investigation and provided a further step toward the integration of local registries into a national one.

## Limitations of the study

The most important limitations of ToRSADE project was based on an anonymous registry that did not allowed cascade family screening and the analysis of variants cosegregation in the family. For the same reason data collection regarding anamnestic symptoms in all potentially sudden death associated diseases such as HCM or Brugada syndrome that is essential (33–38), it was unfortunately not investigated except for survived patients (Table 2). However, these results have led to implement a prospective clinical enrolment protocol, now active at our hospital, in which patients selection is no more anonymous with important clinical consequences for sudden death relatives victims.

## Data availability statement

The datasets presented in this study can be found in online repositories. The names of the repository/repositories and accession number(s) can be found below: https://www.ncbi.nlm.nih.gov/clinvar/, SUB12103611.

## Ethics statement

The studies involving human participants were reviewed and approved by the Local Ethical Committee of Careggi University Hospital, Florence (No. BIO.16.011). The patients/participants provided their written informed consent to participate in this study.

## Author contributions

FG and VS drafted the manuscript, made substantial contributions to the analysis and interpretation of genetic and clinical data, and took responsibility for all aspects of the reliability and freedom from bias of the data presented and their discussed interpretation. MF, VM, RG, and GN made substantial contributions to the analysis and interpretation of data from autopsy and pathological anatomy. MT, NM, and GA made substantial contributions to the analysis and interpretation of clinical data. NM, MT, RC, and BB contributed substantially to the conceptual design of the ToRSADE algorithm. GN and EC made contributions to the analysis and interpretation of data, and critically revised the manuscript for important intellectual content analyzed. EC, RF, and IO reviewed and revised the manuscript. All authors approved the final manuscript as submitted and agree to be accountable for all aspects of the work.

## Footnotes

1
https://www.ncbi.nlm.nih.gov/clinvar/

2
https://gnomad.broadinstitute.org/
