# Supplementary material for: Genetic characterization of juvenile sudden cardiac arrest and death in Tuscany: The ToRSADE registry
Source: Front Cardiovasc Med. 2022 Dec 14;9:1080608. doi: 10.3389/fcvm.2022.1080608 (PMC9795053; doi:10.3389/fcvm.2022.1080608)
Supplement: Supplementary file 1 [file Data_Sheet_1.docx]

Supplementary Material

# Supplementary Data

**Genetic analysis**

Genes analysed in the study. Panel of 174 cardiac genes implicated in cardiomyopathies or channelopathies analysed by NGS. Genomic DNA was automatically extracted from whole-blood samples, using QIAsymphony DSP DNA Kits in combination with the QIAsymphony SP (Qiagen, Hilden, Germany), following the manufacturer’s protocol. Gene libraries were prepared from DNA using an Illumina Nextera TruSight™ Cardio Sequencing Kit (Illumina Inc., San Diego, California, United States). The sequencing step was performed on an Illumina MiSeq system, targeting for 151 bp pair-end reads, and a mean sequencing coverage averaging above 200X. Variant analysis was performed by Local Run Manager and Base Space Variant Interpreter software (Illumina Inc., San Diego, California, United States). Sanger sequencing was used to confirm clinically-relevant detected variants (forward and/or reverse strand). All variants were classified according to the American College of Medical Genetics (ACMG) classification (13) and the Association for Clinical Genomic Science (ACGS) (14,15).

Reanimated subjects received genetic counselling and gave informed consent before performing genetic tests.

Genes Panel:

ABCC9, ABCG5, ABCG8, ACTA1, ACTA2, ACTC1, ACTN2, AKAP9, ALMS1, ANK2, ANKRD1, APOA4, APOA5, APOB, APOC2, APOE, BAG3, BRAF, CACNA1C, CACNA2D1, CACNB2, CALM1, CALR3, CASQ2, CAV3, CBL, CBS, CETP, COL3A1, COL5A1, COL5A2, COX15, CREB3L3, CRELD1, CRYAB, CSRP3, CTF1, DES, DMD, DNAJC19, DOLK, DPP6, DSC2, DSG2, DSP, DTNA, EFEMP2, ELN, EMD, EYA4, FBN1, FBN2, FHL1, FHL2, FKRP, FKTN, FXN, GAA, GATAD1, GCKR, GJA5, GLA, GPD1L, GPIHBP1, HADHA, HCN4, HFE, HRAS, HSPB8, ILK, JAG1, JPH2, JUP, KCNA5, KCND3, KCNE1, KCNE2, KCNE3, KCNH2, KCNJ2, KCNJ5, KCNJ8, KCNQ1, KLF10, KRAS, LAMA2, LAMA4, LAMP2, LDB3, LDLR, LDLRAP1, LMF1, LMNA, LPL, LTBP2, MAP2K1, MAP2K2, MIB1, MURC, MYBPC3, MYH11, MYH6, MYH7, MYL2, MYL3, MYLK, MYLK2, MYO6, MYOZ2, MYPN, NEXN, NKX25, NODAL, NPPA, NRAS, PCSK9, PDLIM3, PKP2, PLN, PRDM16, PRKAG2, PRKAR1A, PTPN11, RAF1, RANGRF, RBM20, RYR1, RYR2, SALL4, SCN1B, SCN2B, SCN3B, SCN4B, SCN5A, SCO2, SDHA, SEPN1, SGCB, SGCD, SHOC2, SLC25A4, SLC2A10, SMAD3, SMAD4, SNTA1, SOS1, SREBF2, TAZ, TBX20, TBX3, TBX5, TCAP, TGFB2, TGFB3, TGFBR1, TGFBR2, TMEM43, TMPO, TNNC1, TNNI3, TNNT2, TPM1, TRDN, TRIM63, TRPM4, TTN, TTR, TXNRD2, VCL, ZBTB17, ZHX3, ZIC3

# Supplementary Figures and Tables

## Supplementary Figure


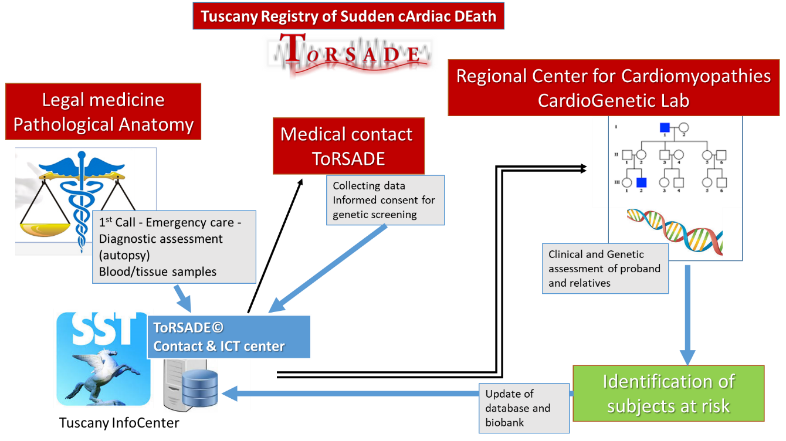


**Supplementary Figure S1.** Flowchart used to generate ToRSADE registry. We outlined an algorithm suitable to monitor, collect, and investigate Sudden Cardiac Arrest (SCA) or Sudden Cardiac Death (SCD) in people aged from 18-50 years, referred to hospitals of the Florentine area. The criteria of inclusion and exclusion were previously defined through a general protocol approved by Local Ethical Committee (No. BIO.16.011).
